# Exosome-Laden Hydrogels: A Novel Cell-free Strategy for *In-situ* Bone Tissue Regeneration

**DOI:** 10.3389/fbioe.2022.866208

**Published:** 2022-04-01

**Authors:** Jinru Sun, Zhifeng Yin, Xiuhui Wang, Jiacan Su

**Affiliations:** ^1^ Institute of Translational Medicine, Shanghai University, Shanghai, China; ^2^ Department of Orthopedics, Shanghai Zhongye Hospital, Shanghai, China; ^3^ Department of Orthopaedics Trauma, Changhai Hospital, Second Military Medical University, Shanghai, China

**Keywords:** hydrogels, exosomes, exosome-laden hydrogels, *in-situ bone tissue engineering*, bone regeneration

## Abstract

*In-situ* bone tissue regeneration, which harnesses cell external microenvironment and their regenerative potential to induce cell functions and bone reconstruction through some special properties of biomaterials, has been deeply developed. In which, hydrogel was widely applied due to its 3D network structure with high water absorption and mimicking native extracellular matrix (ECM). Additionally, exosomes can participate in a variety of physiological processes such as cell differentiation, angiogenesis and tissue repair. Therefore, a novel cell-free tissue engineering (TE) using exosome-laden hydrogels has been explored and developed for bone regeneration in recent years. However, related reviews in this field are limited. Therefore, we elaborated on the shortcomings of traditional bone tissue engineering, the challenges of exosome delivery and emphasized the advantages of exosome-laden hydrogels for *in-situ* bone tissue regeneration. The encapsulation strategies of hydrogel and exosomes are listed, and the research progress and prospects of bioactive hydrogel composite system for continuous delivery of exosomes for *in-situ* bone repair are also discussed in this review.

## Introduction

Currently, bone replacements for skeletal defects are highly required by a majority of patients who suffered accidents or age-related diseases in clinic. It is estimated that more than two million bone grafting procedures are operated per year around the world, with more than a quarter of them operated in the United States (Campana et al., 2014). Moreover, bone grafts need over 600,000 cases in the US caused by cancer and traumatic injuries, which cost about $2.5 billion (Laurencin et al., 2006).

As we all know, autologous bone grafting is always considered as a “gold standard”.

(Ho-Shui-Ling et al., 2018) for clinical treatments of bone defects while its source is limited (Zhang et al., 2019) and secondary surgery caused infection (Laurencin et al., 2006). After that, allografts was developed (Vanderstappen et al., 2015) but the immunological rejection was caused (Dimitriou et al., 2011; Zhang et al., 2019). Therefore, the limitations of autograft and allograft result in alternative bone repair strategies was highly desired and widely developed (Kempen et al., 2009; De Witte et al., 2018).

**FIGURE 1:**
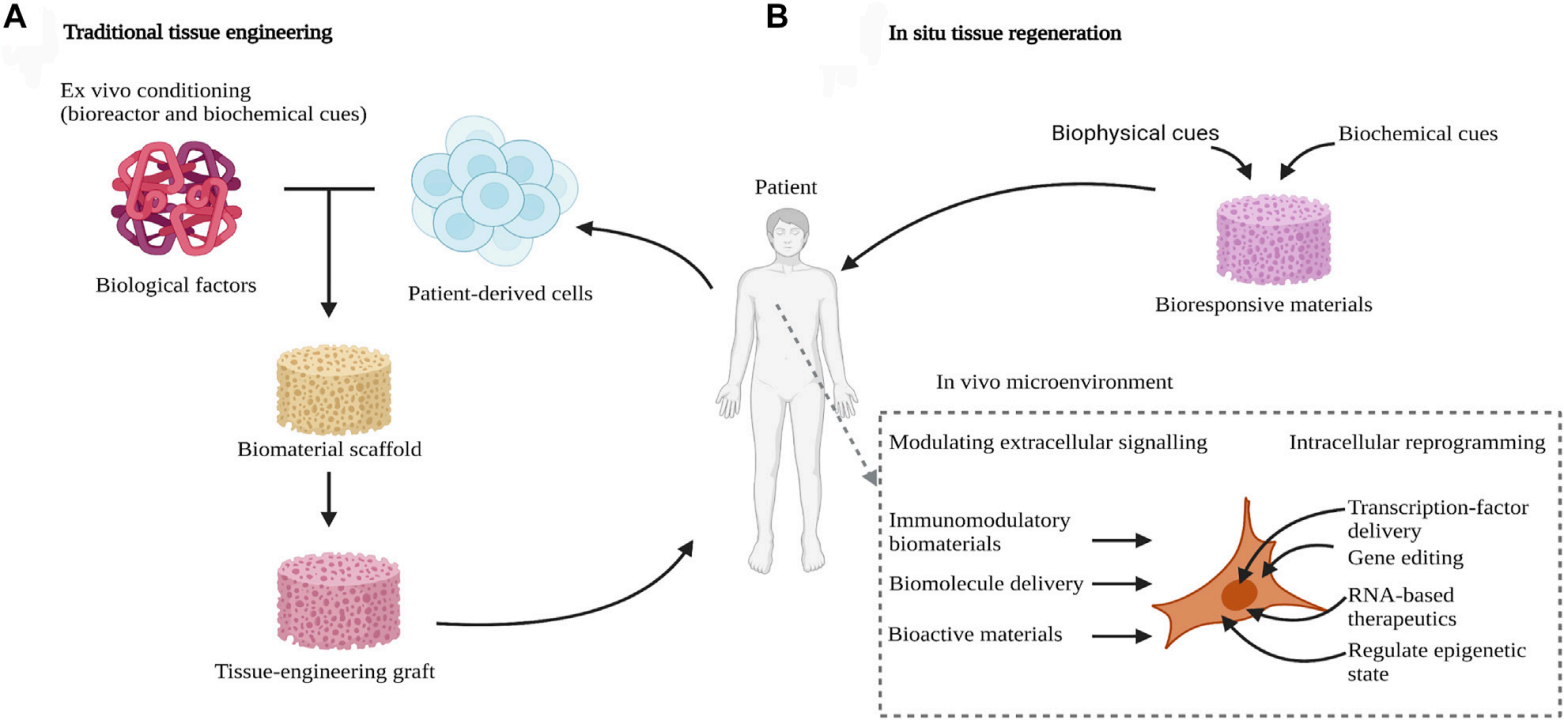
*In-situ* tissue engineering. **(A)** Traditional tissue-engineering approaches require the pre-seeding of engineered scaffolds and *ex vivo* conditioning before implantation into the body. **(B)** *In situ* tissue regeneration uses bioresponsive materials that harness the innate regenerative ability of the body. These materials are loaded with biochemical and biophysical cues to recruit endogenous cells for tissue healing (Gaharwar et al., 2020). Copyright 2020, Gaharwar, A.K., Singh, I. & Khademhosseini.

In recent years, bone tissue engineering strategy, which utilizes the cell culture and functional differentiation *in vitro* to construct bioactive bone-grafts, has been deeply developed for bone regeneration (Figure 1A) (Kempen et al., 2009). Among them, the major elements of bone tissue engineering are seeding cells, growth factors and biomaterial scaffolds (Petta et al., 2016; Yu et al., 2018).scaffold is a crucial factor to bone tissue engineering, which offered the space for cell growth, proliferation and differentiation (Zhang et al., 2010). To promote the three-dimensional attachment, growth and tissue regeneration of cells, the scaffold needs a large specific surface area and interconnected pores (Yu et al., 2018; Zhu et al., 2021). The biomaterials which can be used for fabricating porous scaffolds consist of inorganic ceramic, polymer and metal materials (Yan et al., 2018; Wei et al., 2022). Patients with diabetes mellitus (DM) suffer from poor bone healing ability, the 3D-printed enzyme-functionalized scaffold showed anti-inflammatory and osteogenic effects under diabetic conditions (Yang et al., 2021b). Another study also reported a novel 3D composite scaffold not only triggered the ablation of osteosarcoma *via* high temperature generated by near-infrared II light, but also promoted vascularized bone regeneration *in vivo* by the controlled release of bioactive ions (Sr, Cu, and Si) (Yang et al., 2021a). The scaffold could offer the cells the 3D space, mechanical support and so on. Recently, hydrogels with a 3D network structure, high water absorption and mimicking cell microenvironment have been widely developed. It can be used for the cell encapsulation and ingrowth, thereby promoting their uniform distribution and slightly higher loading densities (Hölzl et al., 2016). Also, bone tissue engineering generally concentrates on fully elastic materials as a result of their superior mechanical strength and stiffness, whereas bone tissue is characteristically viscoelastic. viscoelastic material, which has features such as direct cell behavior and stress relaxation influence, complete with mineralized matrix deposition and osteogenic differentiation (Wang and Yeung, 2017). So, hydrogels with tunable stress-relaxation behavior tend to be a key to direct bone tissue regeneration in non-load-bearing conditions. Mechanically stable 3D constructs can be produced and an excellent biomimetic environment similar to the natural ECM can be provided, in terms of adding hydrogels to robust macroporous scaffolds, while their pores are filled with soft cell-containing hydrogels (Visser et al., 2015; Ovsianikov et al., 2018). However, the limitations of *ex vivo* tissue engineering are noteworthy. This includes donor tissue morbidity, the need for a great number of immune-acceptable cells to fill synthetic scaffold, and the challenges posed by the expansion of large numbers of cells *in vitro*, such as lack and loss of reliable, reproducible cell sources and cellular phenotype (Gaharwar et al., 2020).

Recently, a novel method called *in-situ* tissue regeneration, which leverages the body’s innate regenerative potential, as well as eliminates the need for *ex vivo* cell manipulation, was introduced in (Figure 1B). It has several ways in *in-situ* tissue engineering, such as bioactive cues can be incorporated into biomaterials, to repair the place of injury. *In situ* tissue engineering has advantages over *ex vivo* tissue engineering, because it does not need the process of harvesting cells, thus, reducing regulatory hurdles. In addition, *ex vivo* ways need complex cell culture conditions to obtain functional tissues but *in situ* approaches don’t. Finally, the shelf life of synthetic scaffolds over the cell-laden scaffolds. Therefore, the *in situ* methods have an excellent performance than *ex situ* methods for clinical application (Gaharwar et al., 2020).

In this review, we aim to outline the recent advances of exosome-laden hydrogels for *in-situ* bone tissue regeneration. The advantages of *in-situ* bone tissue engineering compared with traditional tissue engineering were summarized. Moreover, the development and challenges of hydrogels and exosomes for tissue regeneration was elaborated. Besides, the encapsulation strategies of exosome-laden hydrogels are listed, and the research progress and prospects of bioactive hydrogel composite system for continuous delivery of exosomes for *in-situ* bone repair are also discussed in this review.

## Hydrogels Used for *In-Situ* Bone Tissue Regeneration

### Types and Development of Hydrogels

Hydrogelsare three-dimensional (3D) structures formed by physical or chemical cross-linking between hydrophilic polymer chains. It is well known that hydrogels are hydrophilic polymers, with the property of highly-crosslinked water-swollen networks and the ability to swell in water without dissolving. Due to its profound biocompatibility, it could be used in numerous disease treatments as well as play an important role in tissue remodeling (Buwalda et al., 2014). During the biomimetic systems, the hydrogel is a soft material similar to the extracellular matrix, which could generate artificial organs. The material sources of hydrogels can be divided into natural hydrogels and synthetic hydrogels (Zhu and Marchant, 2011). There are four main types of natural polymers including proteins, polysaccharides, protein/polysaccharide hybrid polymers and DNA, could be used to fabricate natural hydrogels. While the polymer types made of synthetic hydrogels were divided into non-biodegradable, biodegradable, and bioactive polymers (Zhu and Marchant, 2011). Natural materials including chitosan, alginate, hyaluronic acid (HA), collagen and gelatin, with the inherent performance of biodegradable and always have integrin binding sites to adhere and coordinate cell responses (Dimatteo et al., 2018). The natural polymers or synthetic polymers used in hydrogels could determine some properties and application of hydrogels. Natural protein polymers are suitable for the preparation of biocompatible hydrogels, while synthetic hydrogels are suitable for various biomedical applications, such as controlled drug release. Moreover, the mechanical property of synthetic hydrogels could be adjustable (Gyles et al., 2017).

### Requirement and Characterization of Hydrogels for Bone Tissue Regeneration

For bone tissue regeneration, hydrogels can be considered as very attractive scaffolds and very promising alternative materials (Bai et al., 2018). And the marked advantage of injectable hydrogels is that they can be implanted in the desired area of tissue through minimally invasive techniques (Staruch et al., 2017). This is because of their suitable properties, including their excellent elasticity, biocompatibility, biodegradability and mechanical properties (Huang et al., 2017; Pishavar et al., 2021). Injectable hydrogels can promote *in situ* tissue regeneration by the way of filling irregular defects.

Also, the different characteristics of hydrogels can be gained through changing the chemical feature of bonds, degree of cross-linking and molecular weight of the polymer (Xue et al., 2022). Moreover, we are facing a huge challenge, for example, the need to combine with the desired characteristics of hydrogels. Because the hydrogel functions explored are sometimes interdependent and sometimes mutually exclusive. For instance, increasing the degree of chemical cross-linking can gain higher stiffness hydrogels. On the contrary, hydrogels with the potential to heal by themselves can be obtained through introducing dynamic cross-linking. Apart from hydrogel structure is required to be adjusted, adding appropriate fillers becomes a strategy to control and manipulate the nano and macro properties of materials (Piantanida et al., 2019). Another factor that should take into consideration is the degradation of hydrogels while designing tissue regeneration scaffolds.

In terms of injectable hydrogels, which have highly concentrated structures including nano-sized pores, micron-level proliferating cells cannot penetrate them without degrading the covalent bonds that bind them together. Therefore, the regeneration of damaged tissue needs to maintain an accurate balance between tissue integration rate and scaffold degradation rate (Deng et al., 2019). On the one hand, slow degradation of materials always leads to an increase in the inflammatory response and can promote fibrosis (Alijotas-Reig et al., 2013). On the other hand, materials that degrade too fast provide insufficient scaffolds to maintain the infiltration and batch arrangement of proliferating cells. To solve these problems, the injectable microporous scaffolds have been designed by some research groups (Bencherif et al., 2012; Griffin et al., 2015), which not only adapt to tissue regeneration but also keep bulk stability. These systems’ widely adoption, provides an ideal design method with scaffold adjustability so that the scaffold can meet the precise physical and chemical requirements of the wound site (Dimatteo et al., 2018; Wu et al., 2021). Moreover, tissue regeneration is closely associated with biomaterials *in situ* degradation. The rate of tissue generation for optimal tissue growth is the same as the biomaterials degradation rate (Gaharwar et al., 2020).

### Application and Prospect of *In-Situ* Bone Tissue Regeneration

**FIGURE 2:**
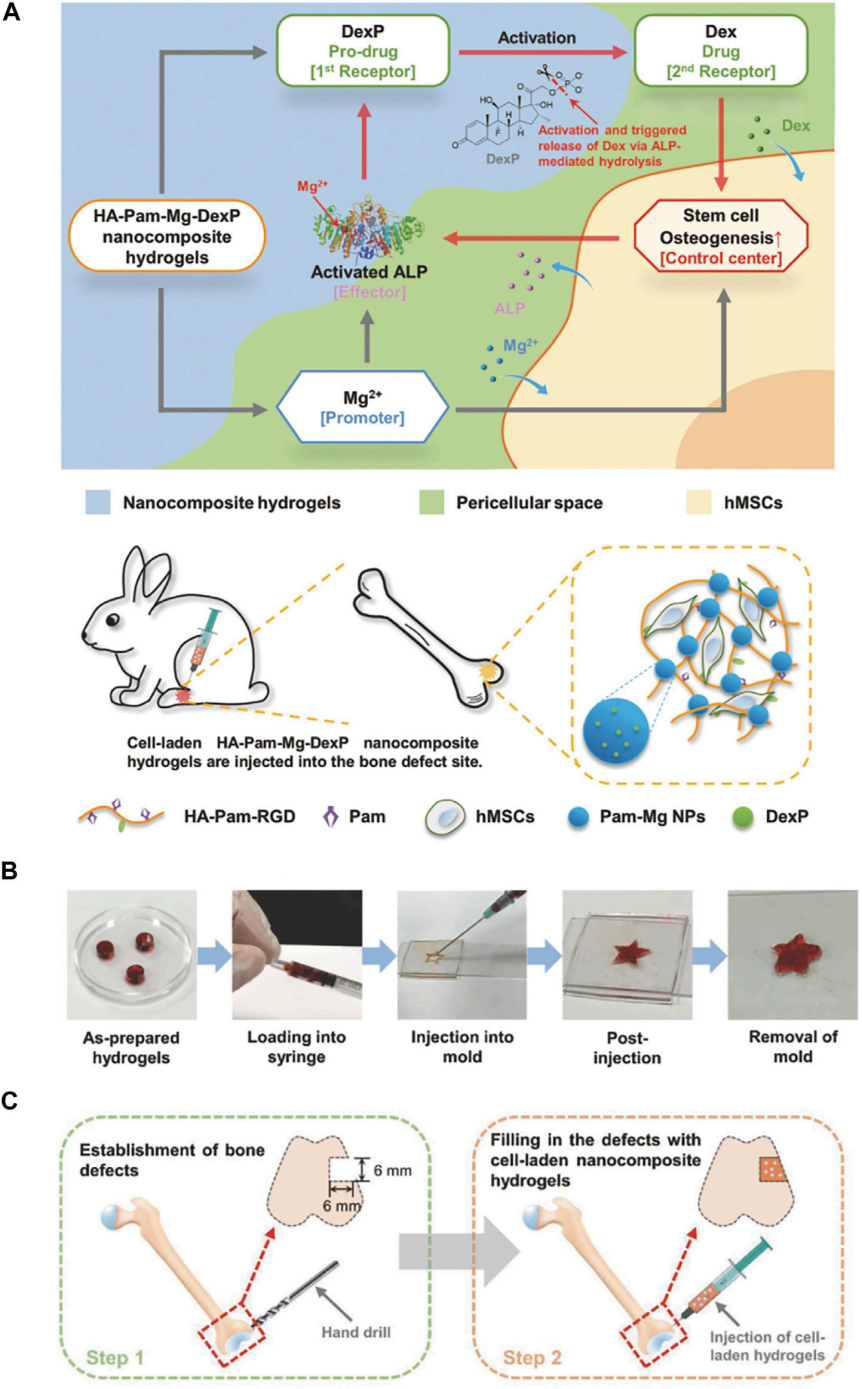
Injectable HA-Pam-Mg nanocomposite hydrogel promotes the healing of bone defects. **(A)** Schematic representation of “smart” hydrogels and injections of hMSC-laden nanocomposite hydrogels promote *in situ* bone regeneration. **(B)** Demonstration of the injectability and formability of nanocomposite hydrogels. **(C)** HA-Pam-Mg nanocomposite hydrogels encapsulating MSCs promote healing of rabbit femur defects (Zhang et al., 2018b). Copyright 2018, WILEY-VCH Verlag GmbH & Co. KGaA, Weinheim.

Zhang and his group fabricated a bioactive nanocomposite hydrogel to regulate the delivery in the local and regeneration-specific release of dexamethasone (Dex). The nanocomposite hydrogel with excellent injection performance and efficient stress relaxation, so it can be easily injected and adapted to irregular bone defects. The release of Mg^2+^ from hydrogel can promote osteogenic differentiation, encapsulate human mesenchymal stem cells (hMSCs), and activate alkaline phosphatase (ALP) (Figure 2). For the sake of promoting hMSCs osteogenesis further, the activated ALP catalyzes the dephosphorylation of Dex phosphate results in releasing Dex from hydrogel quickly. With an emphasis on the bone regeneration rate is better than previous in terms of the positive feedback circuit controlling the activation and release of Dex at the hydrogel implantation sites. The report reveals that injectable nanocomposite hydrogel regulates diverse therapeutic cargoes released in an optimization way and promotes *in situ* bone regeneration through minimally invasive surgery (Zhang et al., 2018b).

**FIGURE 3:**
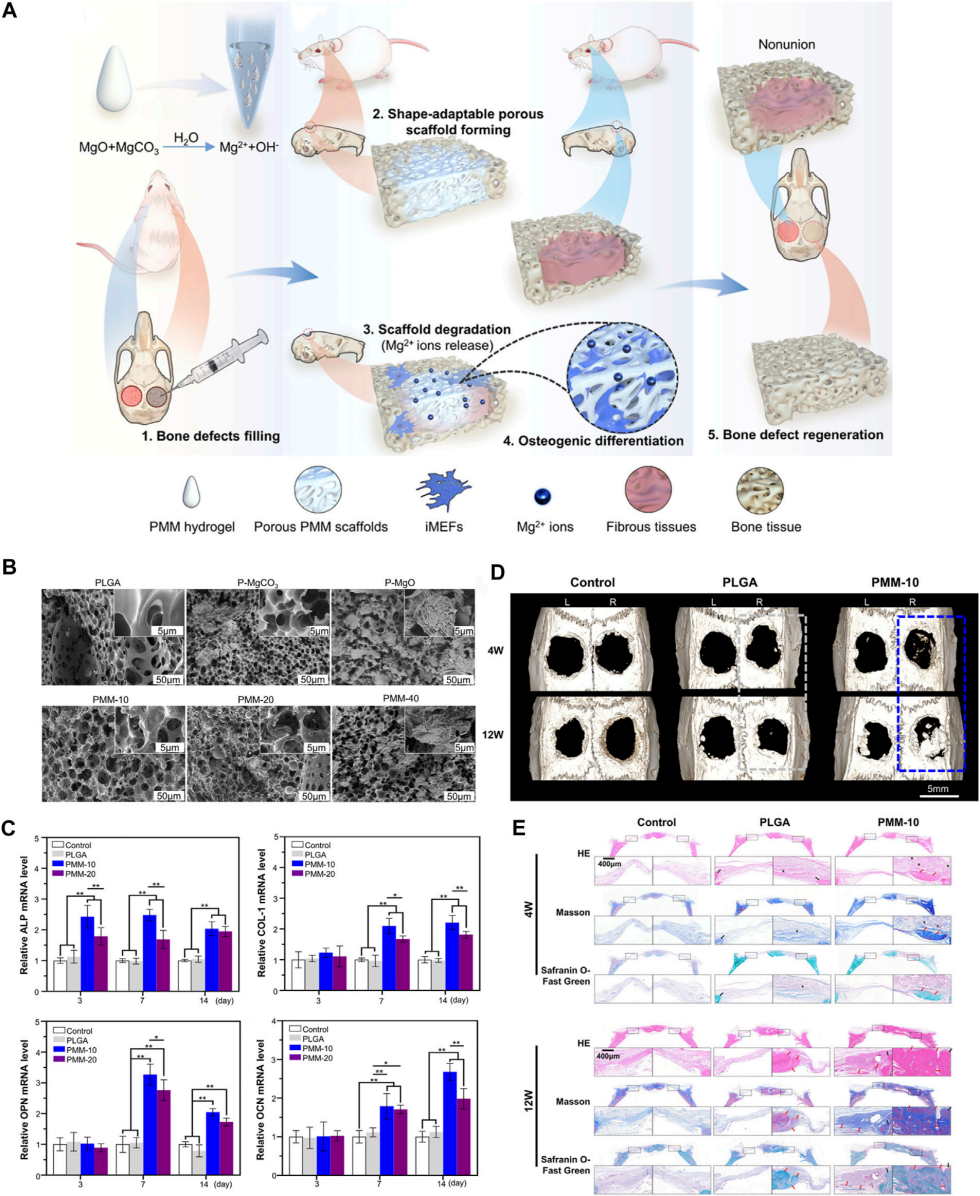
Schematic diagram of MgO/MgCO3@PLGA(PMM) hydrogel promoting bone defect repair. **(A)** Schematic diagram of the mechanism of injectable PMM hydrogel promoting bone defect regeneration. **(B) **SEM images of PLGA and PM scaffolds loaded with MgO/MgCO particles with different weight ratios. **(C)** The relative expression levels of marker genes related to osteogenic differentiation were analyzed. **(D)** Reconstructed 3D micro-CT images of rat crania with the treated defects labeled with rectangular box (gray: PLGA, blue: PMM). **(E)** Histological evaluation of bone defect regeneration using H&E, Masson’s trichrome, and Safranin O-Fast Green staining (*represents residual materials showing the blank area; red arrows indicate new bone, and black arrows indicate host bone) (Zhou et al., 2021). Copyright 2021, American Chemical Society.

Hang reported an excellent injectable MgO/MgCO3@PLGA (PMM) hydrogel to improve bone regeneration. PMM hydrogel not only has good injectable properties, but also can form porous scaffolds *in situ* by solid-liquid transformation, and fill irregular bone defects through its huge shape adaptability. As shown in Figure 3, the injectable PMM hydrogel was investigated for rat calvarial defect repair. Injectable PMM hydrogels can form porous scaffolds *in situ*, through controlled release of Mg^2+^, can meaningfully promote bone regeneration (Zhou et al., 2021). Another study reported an in situ-forming biomaterial, which mixed montmorillonite (MMT) with photopolymerizable methacrylated glycol chitosan (MeGC) hydrogel, could promote bone regeneration. And the nanocomposite hydrogels have great potential to recruit native cells and promote bone formation. Nanosilicate-loaded MeGC hydrogel, which provides a new material design strategy with cell-free and free of growth factors (Cui et al., 2019).

To exploit the potential of hydrogels in various bone regeneration strategies, further research should also focus on developing better compatible nanoparticles (Mehrali et al., 2017). Additionally, one of the biggest challenges still facing bone tissue engineering is that, unlike natural tissues, biomaterials lack the ability to repair themselves (Koons et al., 2020).

## Exosomes: A Cell-free Tissue Engineering Strategy for Bone Regeneration

### Biogenesis and Composition of Exosomes

**FIGURE 4:**
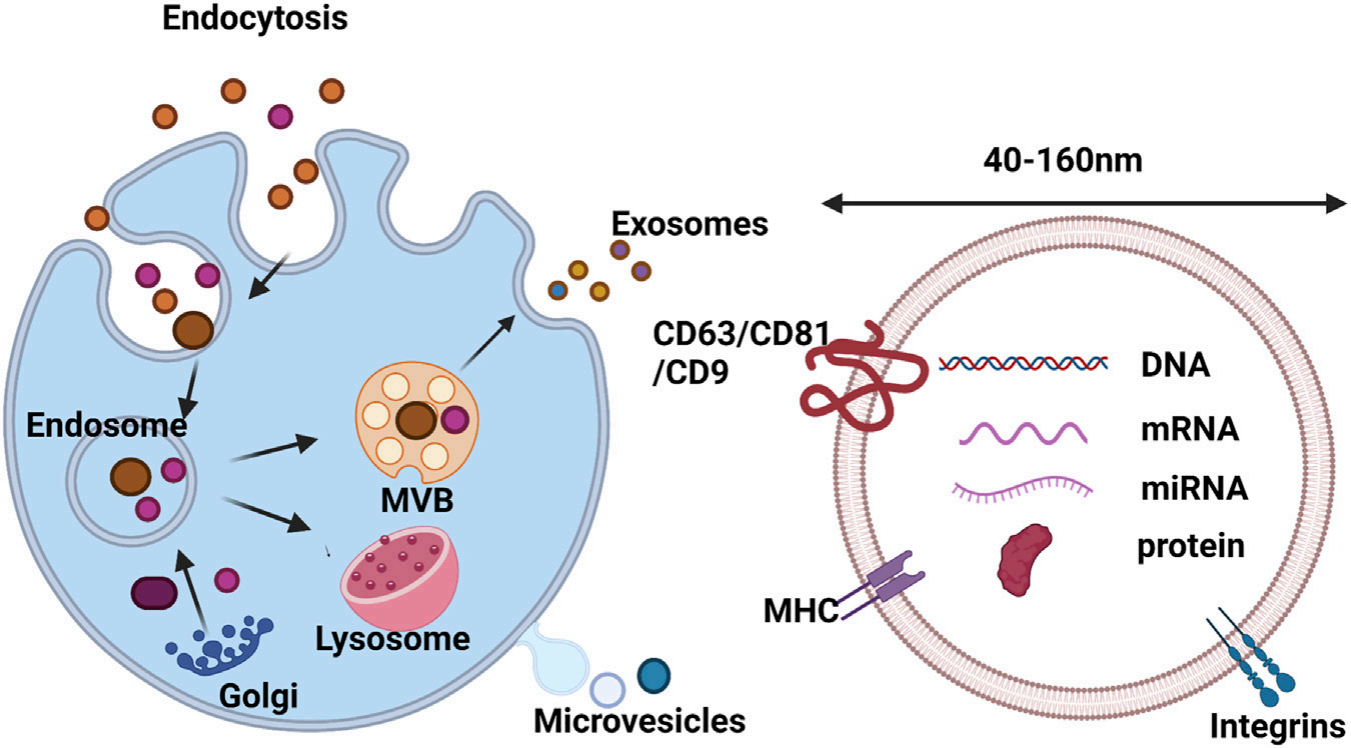
Biogenesis and composition of exosomes. Exosome formation is initiated by invagination of the plasma membrane to form EEs, which fuse to form MVBs. Then, MVBs fuse with the plasma membrane to release exosomes into the extracellular matrix, or fuse with lysosomes for degradation. The composition of exosomes includes lipids, DNA, RNA and proteins (Huang et al., 2021). Copyright 2021, The Royal Society of Chemistry.

Extracellular vehicle (EV) is a phospholipid bilayer spherical structure with substantial dynamic heterogeneity, which is released by almost all mammalian cells and plays a vital role in cell-to-cell communication (Robbins and Morelli, 2014). The exosome is a saucer-shaped vesicle with a diameter of 40–160 nm (Figure 4), which can float in sucrose gradients with a density of 1.13–1.19 g ml^−1^. Plenty of cell types can secret and absorb exosomes, such as endothelial cells, immune cells, tumor cells and mesenchymal stem cells (MSCs) (Huang et al., 2021). Since diverse cells with different characteristic exosomes, this reflects the sorting process of exosomes not just related to the donor cells (van Niel et al., 2006). Some studies have found that both inside and surfaces of exosomes contain cargo, which refers to various proteins and nucleic acids, including DNA, mRNA, miRNA, lipids and small molecules (Mathivanan et al., 2010; D'Asti et al., 2012). It has been demonstrated that some proteins originate from cells or tissue while others are existing in all exosomes by proteomic analysis (Valadi et al., 2007). Generally, the various function of proteins are contained by exosomes such as heat shock proteins (HSP70 and HSP90) not only take part in the stress response but also connect with antigen binding and delivery; tetraspanins such as CD9, CD63, CD81 and CD82, which are involved in cell penetration, fusion and invasion. In addition, in exosome secretion, MVB (Multivesicularbody) formation proteins (Alix, TSG101) and proteins (Annexin and Rab) were found to possess the capacity of membrane transplantation and fusion (Cordonnier et al., 2017). Among the above proteins, some of them are involved in exosome biogenesis, such as fotilin, TSG101 and Alix. These proteins are secreted during plasma membrane spillage, while others exist specifically in exosomes and can be regarded as exosome marker proteins, such as HSP70, TSG101, CD63 and CD81 (Cordonnier et al., 2017; Elkhoury et al., 2020).

Exosomes are involved in the regulation of different signaling pathways in neighboring and distant recipient cells by delivering kinds of biomolecules, including mRNAs, miRNAs, proteins and lipids (Akbari et al., 2020; Hu et al., 2021). As a cell-free biomaterial, exosomes can solve some problems encountered in the clinical application of regenerative medicine, such as the source, quantity and immune rejection of seed cells. Thus, combining exosomes with tissue engineering scaffolds can provide a new generation of scaffold biomaterials that are more suitable for tissue repair (Huang et al., 2021).

### Separation and Extraction Strategies for Exosomes

The limitation of nano-sized and distributing in complex body fluids leads to difficulty to isolate exosomes in high yield (Willms et al., 2018). At the moment, ultracentrifugation is one of the most feasible strategies for exosome isolation due to its high yield, but the high levels of protein aggregates and lipoprotein contamination present in exosome samples prepared by this method are critical for quantitative and functional analysis have a large impact (Li et al., 2017). Since it is impossible for a single way to adapt to diverse sample sources, efforts have been made to explore the different physicochemical and biochemical properties of exosomes. At present, six kinds of methods have been used in exosome separation (Table1), including ultracentrifugation, immunoaffinity capture, ultrafiltration, charge neutralization polymer precipitation, microfluidic technology and size exclusion chromatography. Each method has its unique special advantages and disadvantages (Yang et al., 2020). Exosomes isolated by different methods can usually be identified by detecting their surface morphology, particle size, and surface markers. Commonly used morphological-based identification methods include transmission electron microscopy (TEM) (Manda et al., 2018), scanning electron microscopy (SEM) (Singh et al., 2014), cryo-electron microscopy, and atomic force microscopy (AFM) (Misumi et al., 2018). Identification methods based on exosome size include nanoparticle tracking analysis (NTA) and dynamic light scattering (DLS) (Sitar et al., 2015). Exosome-based identification of various specific or non-specific markers. They contain the same fusion proteins and membrane transport proteins (Annexins, Flotillin, GTPases), Tetraspannins (CD9, CD82, CD81 and CD63) (Zhang et al., 2012). Exosomes derived from MSCs could be stored at −20°C or −70°C, and can maintain biological activity for a long time (Sokolova et al., 2011; Lee et al., 2016).

**TABLE 1:** Current strategies for exosome separation (Yang et al., 2020).

Isolation technique	Advantages	Disadvantages
Sequential ultracentrifugation	• Low cost and	• High equipment requirement
	• Low contamination risk with extra isolation reagents	• Time consuming
		• Labor intensive
	• Suitable for large volume preparation	• Protein aggregation
		• Low portability
Ultrafiltration	• Low equipment cost	• Moderate purity
	• Fast procedure	• Possible loss due to clogging and membrane trapping
	• good portability	
Gradient ultracentrifugation	• High purity of products	• Lower volume process ability
	• Allowing separation of subpopulation of exosomes	• High equipment requirement
		• Time consuming
		• Labor intensive
		• Low portability
Size-exclusion chromatography	• High purity	• High device costs
	• Fast preparation	• Additional method for exosome enrichment is required
	• Keep native state of exosomes	
	• Good reproducibility	
	• Potential for both small and large sample capacity	
Immunoaffinity capture	• Suitable for separating exosomes of specific origin	• High-cost antibodies
	• High-purity exosomes	• Exosome markers must be optimized
	• Easy to use	• Low processing volume and yields
	• No chemical contamination	
Microfluidics-based techniques	• Highly efficient	• Low sample capacity
	• Cost-effective	
	• Portable	
	• Easily automated and integrated with diagnosis	
Polymer Precipitation	• Easy to use	• Contaminants of protein aggregates, other extracellular vesicles and polymeric contaminants
	• Using ordinary equipment	• Extended processing time
	• Suitable for both small and large sample volume	• Require complicated clean-up steps
	• High efficiency	

### Recent Advances of Exosomes for Bone Tissue Regeneration

Recently, compared with other cell-based therapies, the secretion of MSCs have received considerable attention as a regeneration tool (Liu et al., 2017). First discovered in the 1960s, MSCs were originally described as spindle cells derived from bone marrow, which regulate the quiescence and self-renewal of hematopoietic stem cells *via* the release of paracrine factor (Pluchino and Smith, 2019). These cells with the feature of heterogeneous, apart from bone marrow, have been successfully isolated from the placenta, amniotic fluid, adipose and other tissues. Exosomes, which are derived from MSCs, have a vital influence on the function of endothelial cells and promote tube formation and thus play a role in angiogenesis and vascular network maturation (Li et al., 2016). It is easy to isolate bone marrow mesenchymal stem cells from adult tissues and have great expansion ability *in vitro*. Several evidence has been shown that MSCs with an outstanding therapeutic role in plenty of diseases (Khayambashi et al., 2021).

There is no doubt that the MSC-based tissue engineering method is an innovative strategy for clinical treatment (Lin et al., 2017). Nevertheless, it has been found that they are instability and with the potential to form cancer (Carson et al., 2006). These findings lead the research community to reconsider the biosafety of stem cell therapy. With the development of cell-free therapies, exosome has gradually become a tool for tissue repair, which is better than traditional stem cell therapy because it conquers risks and limitations.

Zhang et al. (Zhang S. et al., 2018) reported that MSC-derived exosomes with the potential to repair osteochondral defects through a way that contains increased migration, matrix synthesis and proliferation, decreased apoptosis and regulated immunoreaction. Cui et al. (Cui et al., 2016) reported that mineralized osteoblasts derived exosomes affected the miRNA profile of recipient bone marrow cells, thus promoting differentiation into osteoblasts. Owing to a change in miRNA profile, the expression of Axin 1 was inhibited, whereas the expression of *ß*-catenin was increased as well as the Wnt signaling pathway was activated (Gu et al., 2021).

Studies have shown that exosomes from MSCs with similar functions to MSCs, including tissue regeneration and repair, inhibition of inflammation, regulation of immunity and so on (Askenase, 2020). Some advantages of using exosomes for tissue regeneration rather than MSCs are as follows. First of all, the immune risks associated with stem cell transplantation are avoidable. And exosomes cannot self-replication without the potential to form endogenous tumors (Lener et al., 2015). A report showed that a spinal cord–injured patient, who transplanted olfactory mucosal stem cells, formed tumors at the injured site (Dlouhy et al., 2014), emphasizing stem cell therapy with potential risks. Second, compared with MSCs, exosomes can be stored for a longer time and can be used more conveniently. Third, differ from exosomes, MSCs are too big to circulate through capillaries. Especially, exosomes can promote lung repair by entering the lungs after infecting Corona Virus Disease 2019 (COVID-19) (Askenase, 2020). Finally, in contrast with MSCs, the biogenesis and functional characteristics of exosomes can be defined more correctly. The function of MSCs can be reprogrammed by environmental factors, but not exosomes (Lener et al., 2015). All of these advantages make MSC exosomes can be administered easily and treat kinds of diseases safe (Shiue et al., 2019).

**FIGURE 5:**
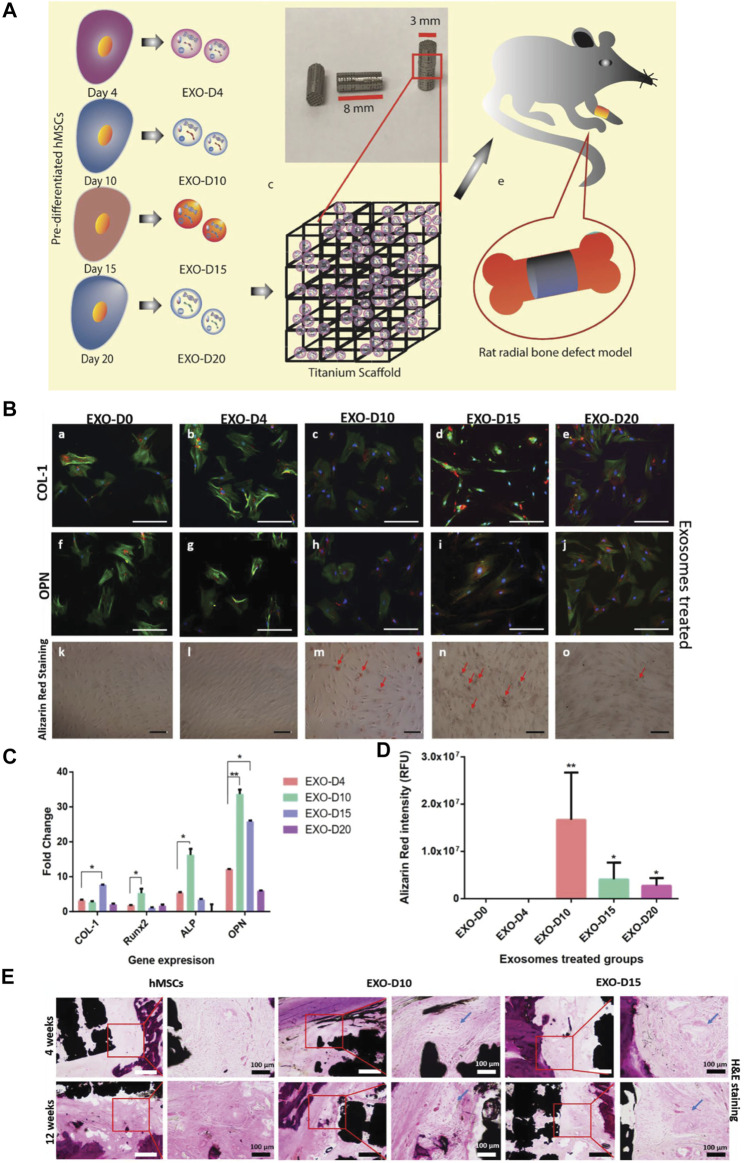
Overview of stem cell-derived exosomes for bone tissue regeneration. **(A)** Exosomes from hMSCs were isolated and complexed with Ti scaffolds and implanted into the radial defect of the rat. **(B)** Immunofluorescence staining of osteogenic markers (COL-1[a-e]; OPN[f-j]) in hMSCs showing osteogenic exosome-induced osteogenic differentiation. **(C)** Real-time PCR of osteogenic markers (COL-1, Runx2, ALP, and OPN) showed exosome-induced osteogenesis of hMSCs. **(D)** Intensity of Alizarin Red after induction in various exosomes. **(E)** H&E staining demonstrated new bone formation *in vivo* (Zhai et al., 2020). Copyright 2020 The Authors. Published by Wiley-VCH GmbH.

A study reported that exosomes secreted by human mesenchymal stem cells (hMSCs) could induce osteogenesis of hMSCs through osteogenic pre-differentiation at different times, and the extracted exosomes were combined with 3D printed titanium alloy scaffolds for cell-free bone regeneration (Figure 5). The results showd that the bone tissue regeneration efficiency of cell-free exosome scaffolds was comparable to that of hMSC-seeded scaffolds, so replacing stem cells with osteogenic exosomes secreted by pre-differentiated stem cells was expected to become a new cell-free bone regeneration pathway (Zhai et al., 2020).

### Limitation and Prospect of Exosomes for *In-Situ* Bone Regeneration

Although we all know the benefits of exosomes, the shortcomings of delivering a therapeutic dosage of exosomes, peculiarly *via* systemic injections, may over their advantages (Riau et al., 2019). The common ways to administer exosomes are intravenous, subcutaneous, and intraperitoneal injections. When it comes to the exosomes’ biological effects, the crucial factor that must be considered is the target cell, which could internalize exosomes through endocytosis, if not, exosomes will enter the blood circulation and be quickly removed. Since the short half-life exosomes possess, which exist only 2–4 min (Saunderson et al., 2014), and will be quickly removed from the blood vessels. Then exosomes will enter the organs (Schiffelers et al., 2000). For example, exosomes isolated from B16-BL6 mouse melanoma cells rapidly disappeared after intravenous injection with a half-life of 2 min (Takahashi et al., 2013). Further study has been demonstrated that after 2 h systemic injection, exosomes can be found in the liver, lung, spleen and gastrointestinal significantly (Takahashi et al., 2013; György et al., 2015). Generally, these exosomes are mainly phagocytosed by macrophages in the spleen or liver (Huang et al., 2021). On the one hand, the injection ways of exosomes like direct intravenous, subcutaneous and intraperitoneal injection, can cause a macrophages response in the reticuloendothelial system, leading to rejection. When applied systemically or locally (skin or eye), exosomes have shown a short half-life after interacting with sweat, tears and the epithelial barrier (Riau et al., 2019). On the other hand, it is difficult to purify and produce exosomes on a large scale due to the demand for consistency of nanometer-sized exosomes by the costly protocols (Riau et al., 2019).

To solve this problem, the exosome therapy research with emphasis on the combination of exosomes and biomaterials. The durability and stability of exosomes can increase significantly while combined with diverse biomaterials as scaffolds. Furthermore, the ideal biomaterial should with the capacity of maintaining the bioactivity of exosomes and controlling the release kinetics of exosomes in terms of the expected release schedule. In addition, the characteristic of biomaterials must be taken into consideration, which can influence the efficiency of loading or releasing exosomes. Thus, when it comes to materials design, porosity is a fundamental element that needs to be emphasized, which can promote substance transport in the injured tissue owing to highly connected porous networks. Through the micro or nanoscale porosity can release bioactive agents, move gasses, nutrients, and waste products better than materials of other sizes. Tissue engineering biomaterials as similar to the natural ECM, which can supply migration, growth and survival of MSCs with a scaffold. As biomaterials for bone tissue repair, with proper stability and integrity, and have appropriate stiffness and mechanical properties like bone tissue look necessary. Undoubtedly, the scaffold needs the potential to be biodegradable, and the degradation rate should match the regeneration of new tissue so that the scaffold could be replaced. When cleavable groups need to incorporate into the scaffold, the rate of degradation should be controlled primarily. What important most is the biocompatibility of biomaterial, the potential to perform without causing adverse host reactions, and it should not accumulate in the body, thus the biomaterial and degradation products should be bio-absorbable (Safari et al., 2021).

As we all know, natural ingredients obtained from biological sources with inherent biocompatibility can be well applied in the body and can also be degraded by enzymatic cleavage easily. However, synthetic biomaterial with more fantastic functions and structures. The biomaterials will be more universal if change the molecular composition gain new properties and optimize the existing properties and so on. Beyond that, kinds of membranes, nanoparticles and hydrogels have been used to promote the controlled release of bioactive molecules in tissue repair (Ding et al., 2014; Xu et al., 2015; Liang et al., 2019; Safari et al., 2021).

Without encapsulation, exosomes can be cleared from the body through fluids at a quick speed (Riau et al., 2019). Thus, delivering exosomes needs a more powerful way to avoid clearance by the host (Khayambashi et al., 2021).

## Exosomes Laden Hydrogels for Inducing *In-Situ* Bone Regeneration

### Hydrogel as a Vehicle for Exosome Delivery

It is a popular choice to apply hydrogels as a delivery system and scaffold materials owing to hemostatic ability, antibacterial activity, injectability, tissue adhesion, self-healing and so on (Li et al., 2015; Huang W. et al., 2016; Lokhande et al., 2018; Safari et al., 2021). Hydrogel encapsulated exosomes can protect them without degradation and supply therapeutic effects with persistent exosomes delivery (Riau et al., 2019). Currently, the local continuous drug delivery of exosomes is available through hydrogels as carriers. For instance, previous studies reported that the MSC-EVs combined with chitosan and silk fibrin-synthesized hydrogels showed a sustained release and long-term wounding healing for up to 2 weeks (Shi et al., 2017). The property of hydrogels, such as hydrophilic and cross-linking behavior, have promoted the capability of controlled drug release. Besides, it has demonstrated that hydrogels with important effects on the fields of bone formation, angiogenesis, immunology and oncology (Mantha et al., 2019). It has been studied that the purified, unformulated exosomes biodistribution in animal models. The vary of administrations including intravenous, subcutaneous, intraperitoneal, intranasal and retro-orbital, were used to evaluate disposal and exosome kinetics *in vivo* (Zhang et al., 2018a).

In comparison with stem cells, exosomes with more advantages in tissue regeneration can maintain biological activity and are highly stable for some time. Additionally, exosomes have the capability for targeting organs, initiating tissue regeneration, and protecting plenty of bioactive ingredients without degradation (Lou et al., 2017). Owing to exosomes do not have self-replicating characteristics, exosomes can reduce the danger of iatrogenic tumor formation and can reduce the formation of embolism when MSCs are injected. However, purified unformed exosomes can be cleared from the host at a short period after being absorbed by the reticuloendothelial system (Conlan et al., 2017). To conquer these limitations, hydrogels with the property of degradation can play a crucial role in protecting exosomes and take for a carrier and delivery depots of exosomes in the entry site so that a more durable therapeutic effect will obtain.

In addition, the high concentration of therapeutic molecules involved in exosomes can be delivered locally when exosomes are combined with hydrogels and applied near or in the target tissue site (Riau et al., 2019). Because of the structural or physicochemical characteristics of the hydrogel, the degradation rate of the hydrogel matrix can be adjusted, and the release and functional characteristics of the embedded exosomes can be controlled.

Furthermore, biodegradable hydrogel should be taken into consideration as an outstanding candidate for exosomes encapsulation in plenty of treatments, because they have the ability of biocompatible and are similar to the intracellular matrix. These advanced hydrogel-exosome formulation platforms could offer special formations to tissue engineering, for example, bone repair (Liu et al., 2019). To a certain degree, the treatment effect of exosomes depends on the design and function of hydrogels (Pishavar et al., 2021).

### Approaches of Hydrogel-Exosome Encapsulation

At present, there are several methods to transport exosomes to target tissues and organs, which can be divided into systematic and local. Systemic ways of administration include intravenous, oral, intranasal, intraperitoneal, and subcutaneous, while local administration can be realized by directly loading exosomes suspension or loading exosomes into biomaterials (Pinheiro et al., 2018; Alqurashi et al., 2021). The therapeutic benefit process of using exosomes is enhanced by the use of hydrogels in bone tissue engineering. Generally, there are three ways to encapsulate exosomes into a hydrogel matrix (Riau et al., 2019).

**FIGURE 6:**
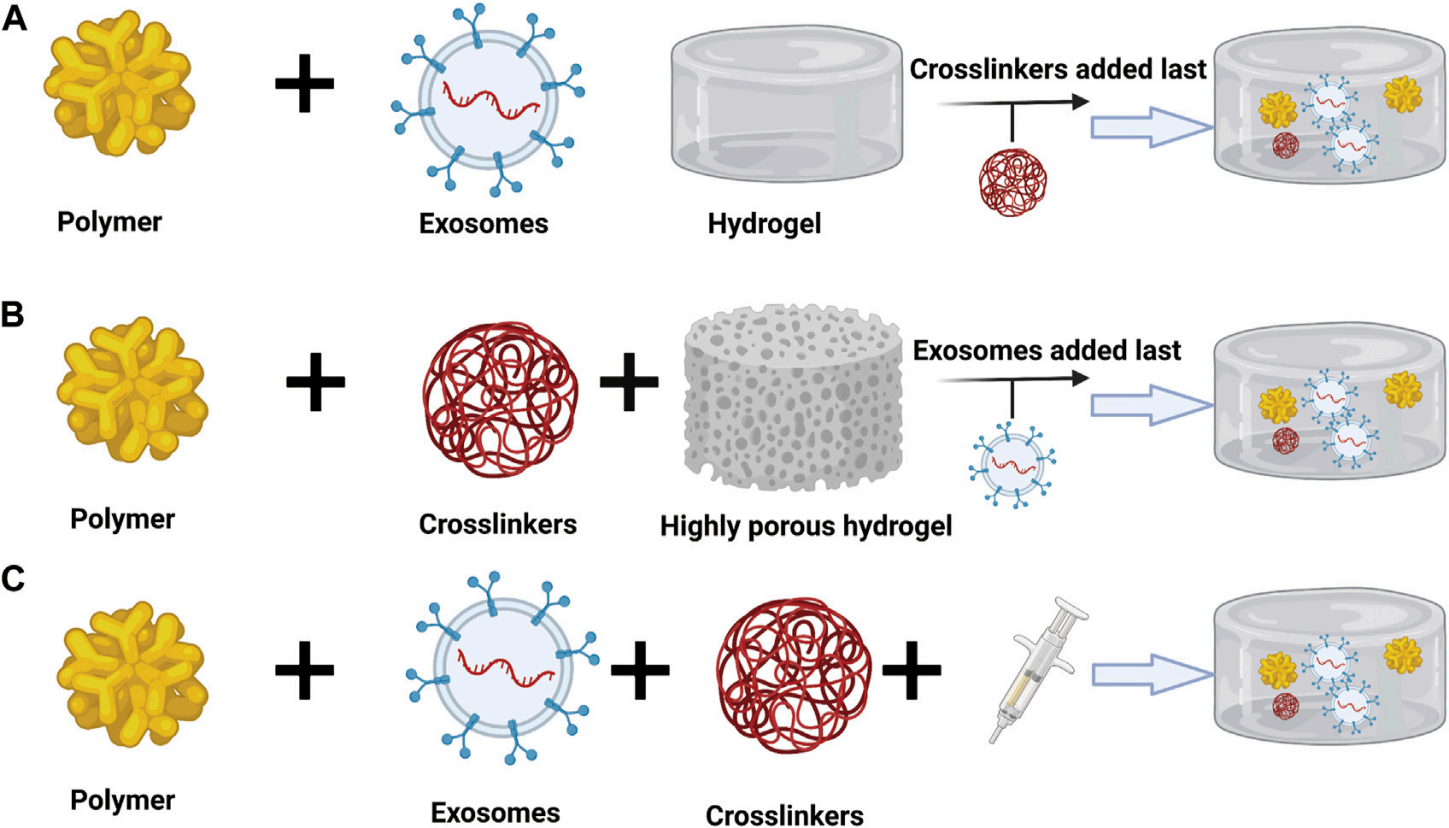
Approaches to encapsulate exosomes in hydrogels: **(A)** Combining the exosomes with polymers followed by the addition of crosslinkers to induce gelation. **(B)** Physical incorporation of the hydrogels, or the “breathing” technique. **(C)** Mixing of the exosomes with both the polymers in solution and crosslinkers simultaneously (Khayambashi et al., 2021). Copyright 2021, MDPI, Basel, Switzerland.

The first means is that exosomes are combined with the polymer, and then a cross-linking agent is added to induce gelation (Figure 6A). Studies have reported this method, which uses hyaluronic acid (HA), gelatin and heparin to form a polymer. Exosomes derived from bone marrow stem cells are incorporated into this polymer, and polyethylene glycol diacrylate (PEGDA) is used as the gelling agent of the system (Ghosh et al., 2005; Qin et al., 2016). This method is based on the active precursor for covalent cross-linking. Since this technology provides hydrogels with adjustable properties, controllable mechanical properties and degradation rates, it is an attractive strategy for exosomes and cell encapsulation (Nicodemus and Bryant, 2008). Nevertheless, a universal problem that exists is that when new compounds are added, such as cross-linking agents, they may be potentially cytotoxic to biomolecules. The advantage of this method is the use of macromonomers, which are usually derived from biocompatible polymers (Khayambashi et al., 2021).

The second way is the physical combination of hydrogel or “breathing” technology (Figure 6B). This technique has two main steps. First, the water in the hydrogel is removed by putting the swollen hydrogel in a solvent. The second step is to put the hydrogel in an aqueous solution containing exosomes so that the porous hydrogel is absorbed into the exosomes (Thomas et al., 2009). According to the principles of smart hydrogels, the method has been developed. That is, the hydrogel will form a swelling structure when in water, and the hydrogel will even collapse and undergo a phase change in a low-polar solvent (Shipway and Willner, 2001). Nevertheless, to use the method, the pore size of the hydrogel needs to be adjustable, and there is no doubt that the pore size needs to be larger than the encapsulated exosomes. Once inside the cell, loosely attached exosomes will effusion when exposed to the target (Thomas et al., 2009).

The last method is to mix the polymer and the crosslinker in the solution with the exosomes at the same time (Figure 6C). A study used this method, which resulted in situ gelation, enabling targeted delivery of exosomes to the site of action. They used fat-derived exosomes and peptides for wound healing (Wang et al., 2019). In general, this strategy requires the use of a dual-cavity syringe, which has the ability to inject the hydrogel with exosomes directly into the defect site (Ghosh et al., 2005; Riau et al., 2019). There are a variety of mechanisms that can be used for *in-situ* gelation, such as ultraviolet radiation, ion exchange, pH changes, and temperature changes (Ruel-Gariépy and Leroux, 2004). This strategy is very significant in filling the critical size defects of complex shapes, allowing the combined biomolecules to have good viability. This type of injectable scaffold has the required inherent tissue properties, so it can work alone without external induction (Sargeant et al., 2012; Khayambashi et al., 2021).

### Advances and Development of Exosome-Laden Hydrogels for *In-Situ* Bone Regeneration

The interaction between exosomes and biological materials determines the effective concentration of exosomes in biological materials. Electrostatic interaction and biologically active adhesion are the main ways to combine exosomes with biological materials. The mutual attraction or repulsion between exosomes and biological materials depends on the negatively charged phospholipid membrane of exosomes and the charged residues of glycocalyx (Gerlach and Griffin, 2016). For example, a cation delivery system containing chitosan hydrogel retains exosomes through electrostatic force. The exosomes derived from MSC express adhesion molecules, for example, CD44 and *a* integrins. Therefore, exosomes have biological activity on extracellular matrix ingredients, for instance, type I collagen, fibronectin and hyaluronic acid and so on (Huang C.-C. et al., 2016; Brennan et al., 2020; Safari et al., 2021).

**FIGURE 7:**
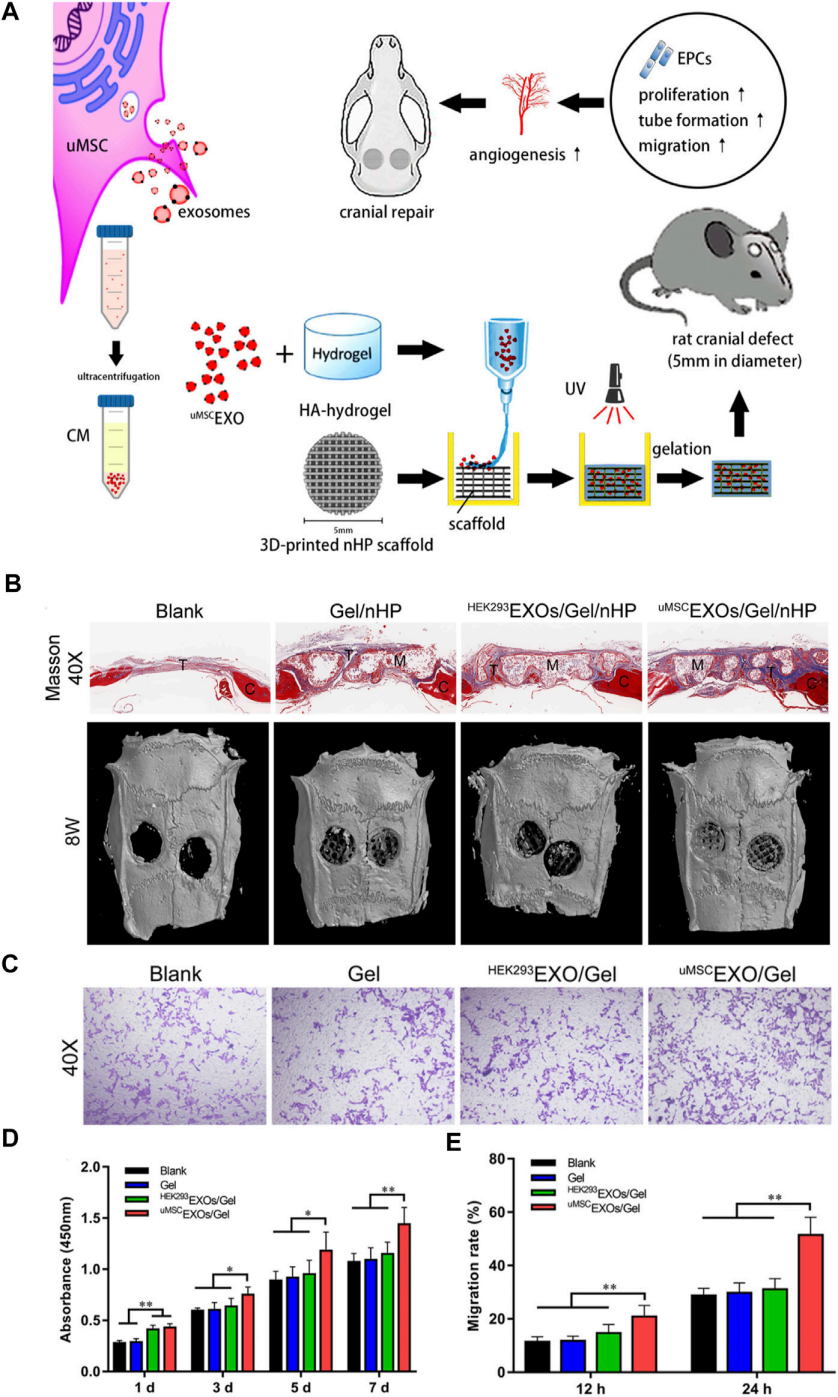
Schematic diagram of ^uMSC^EXOs combined with HA-Gel and nHP scaffolds to promote the repair of skull defects. **(A)** Hydrogel composite exosomes scaffold promotes angiogenesis to repair critical size skull defects in rats. **(B)** The micro-CT scan image and quantitative analysis of the Masson’s trichrome (40 × ) image after 8 weeks of repairing the critical size skull defect *in vivo* in the rat, showing the skull defect area. **(C)** Transwell migration analysis of endothelial progenitor cells in different treatments. **(D)** CCK-8 shows that ^uMSC^EXOs can promote EPCs proliferation. **(E)** Quantitative analysis of the migration rate (Zhang et al., 2021). Copyright 2021, American Chemical Society.

Zhang has reported that a nanoparticle composite was prepared by encapsulating umbilical MSC-derived exosomes (^uMSC^EXOs) in hyaluronic acid hydrogel (HA-Gel), and combining them with nanohydroxyapatite/poly-ε -caprolactone (nHP) scaffold combined to repair rat skull defects (Figure 7). The methods of imaging and histological evaluation have shown that the ^uMSC^EXOs/Gel/nHP composite material significantly promotes bone regeneration *in vivo*, and ^uMSC^EXOs may be essential in the bone repair pathway. In addition, *in vitro* experiments have shown that ^uMSC^EXOs have the potential to make endothelial progenitor cells (EPCs) proliferate, migrate, and angiogenesis, but have little effect on the osteogenic differentiation of bone marrow mesenchymal stem cells. It cannot be ignored that mechanism studies have shown that exosomal miR-21 is a potential intercellular messenger, which promotes angiogenesis by up-regulating the NOTCH1/DLL4 pathway. In summary, the results of the study show a way to use exosomes to repair bone defects, which may be regulated by the miR-21/NOTCH1/DLL4 signal axis (Zhang et al., 2021).

**FIGURE 8:**
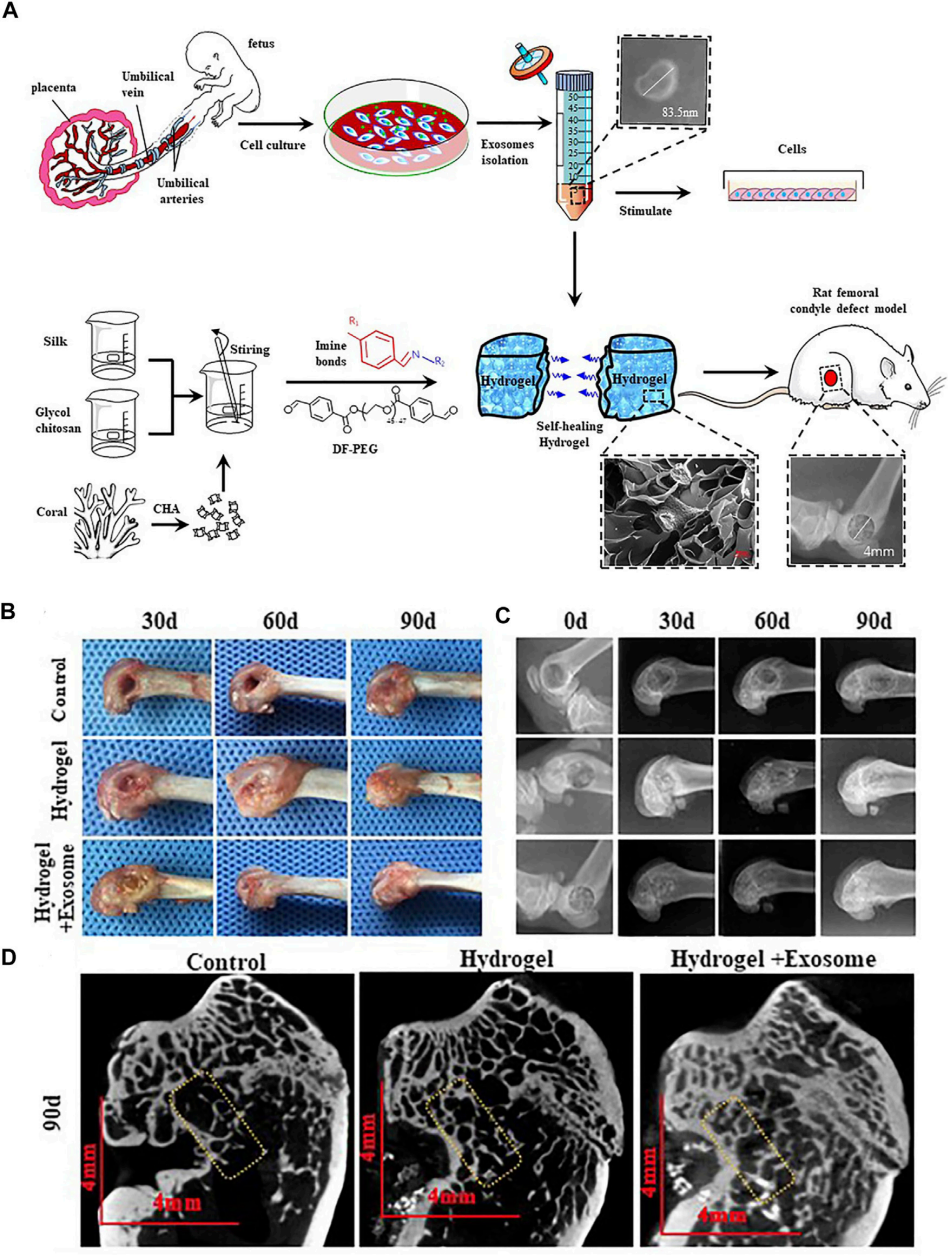
HucMSC-derived exosomes combined with CHA/SF/GCS/DF-PEG hydrogel for the treatment of femoral condyle defects in rats. **(A)** The separation and identification of HucMSC-derived exosomes and the preparation of CHA/SF/GCS/DF-PEG hydrogel are used for testing on SD rats with induced femoral condyle defects. **(B)** 0, 30 and 90 days after implantation of CHA/SF/GCS/DF-PEG hydrogel or CHA/SF/GCS/DF-PEG hydrogel combined with hucMSC-derived exosomes, gross observation **(C)** X-ray **(D)** Micro-CT imaging results (Wang et al., 2020). Copyright 2020, Frontiers in Bioengineering and Biotechnology.

Another research has been reported that a self-healing coralline hydroxyapatite (CHA)/silk fibroin (SF)/glycol chitosan (GCS)/difunctionalized polyethylene glycol (DF-PEG) hydrogel was successfully prepared (Figure 8), which has perfect comprehensive properties. Moreover, it is expected to be an excellent material that will be used in bone graft. The better bone repair effect will be if add hucMSC-derived exosomes to this hydrogel (Wang et al., 2020).

## Future and Prospects

In the past few decades, bone tissue engineering has gradually developed, especially in the past few years due to the rise of *in-situ* tissue engineering. In this context, the composite of various types of hydrogels and nanoparticles that are similar to natural extracellular matrixes has obvious advantages in the treatment of bone tissue repair. There is no doubt that the evaluation of the main properties of hydrogel composite nanoparticles, such as biocompatibility and biodegradability, is necessary. In addition, their interaction with surrounding tissues is also an important factor that must be considered. Seed cells are widely used as a key element in regenerative medicine. For example, mesenchymal stem cells (MSCs) derived from various sources have good prospects in clinical research as cell-based therapies. As the core of tissue repair, seed cells are widely used in various fields of regenerative medicine. However, the use of stem cells for treatment always has problems such as low cell survival rate and immune rejection. Therefore, the use of stem cells for tissue regeneration has safety issues that cannot be ignored (Hofer and Tuan, 2016). And most of the therapeutic benefits of MSCs come from the release of paracrine factor exosomes with anti-inflammatory activity. The exciting discovery of exosomes contributes to cell-free therapy in tissue regeneration. Exosomes are nanoscale extracellular vesicles that contain biologically active molecules such as RNA and proteins; therefore, exosomes have similar functions to parent cells. Although the size of exosomes is similar to liposomes, naturally derived exosomes have many natural advantages over other nanoparticles. Naturally derived exosomes have outstanding biocompatibility, biodegradability, low toxicity and immunogenicity. The major limitations of exosome extraction was the purity and mass production, which restricted the wide clinical application (Aheget et al., 2020). Moreover, exosomes had their own inherent limitations including low targeting capacity, low circulating half-life and low concentration of functional molecules, which affected the clinical effectiveness (Jafari et al., 2020). The separation of exosomes is also a key issue. At present, ultracentrifugation is the most common method for extracting exosomes, but it also has disadvantages such as lipoprotein contamination. If we want to make progress in the field of exosomes research, we must develop efficient exosomes separation technology. A large number of studies have shown that the compounding of exosomes and hydrogel can improve the stability of exosomes and provide a continuous treatment environment for tissue defects. In addition, it contributes to maintaining the content of exosomal protein and miRNA in the body. However, many current treatment strategies to promote bone tissue repair also have shortcomings. These limitations include how the biomimetic scaffold is optimized to be like natural tissues and how bioactive molecules can deliver and maintain activity more efficiently. With the comprehensive disclosure of exosomes and their functions, the combination of exosomes and hydrogels will have more applications that cannot be ignored in clinical practice.
